# Isobaric Molecular Dynamics Study of Liquid Film Boiling

**DOI:** 10.1021/acs.langmuir.5c06528

**Published:** 2026-02-23

**Authors:** Avik Saha, Omar K. Matar

**Affiliations:** Department of Chemical Engineering, 4615 Imperial College of London, London SW7 2AZ, U.K.

## Abstract

This study employs piston-based isobaric molecular dynamics simulations to analyze the liquid film beneath nucleating bubbles. The isobaric piston system is validated across different liquid and gaseous systems, and the Lennard-Jones (L-J) and repulsive-only force fields are applied to evaluate their suitability for boiling simulations. Using this system, the effects of surface wettability and pressure on nonevaporating films are explored. Additionally, a novel boundary force field is developed to mimic the behavior of boundary molecules fully immersed in the liquid pool, enabling the simulation of boiling in a thick liquid film. The interfacial thermal resistance and accommodation coefficient are calculated based on the measured depletion rate of the liquid film with different thicknesses, providing valuable insights into the fundamental mechanisms of boiling.

## Graphical abstract

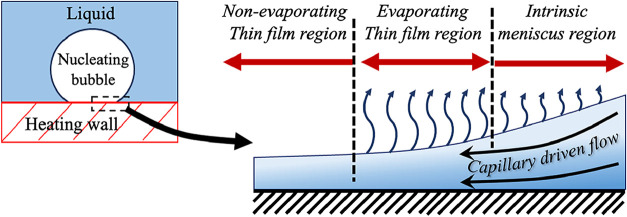

## Introduction

Boiling is one of the most effective heat transfer mechanisms, widely utilized in applications such as power generation, refrigeration, and thermal management. Due to its high heat transfer efficiency, boiling has been extensively studied over the years. Among its various regimes, nucleate boiling has received significant attention because it provides high heat flux at relatively low wall superheat. Despite decades of research, modeling nucleate boiling remains a considerable challenge. Most current approaches rely on empirical formulations derived from experiments. While these formulations can predict boiling behavior under specific surface and flow conditions, their applicability is limited, and a generalized, physics-based model remains elusive.

To develop such a universal model, it is essential to understand the fundamental mechanisms underlying nucleate boiling, which remain poorly explored. Nucleate boiling is a complex, multiphysics phenomenon involving heat and mass transfer, bubble nucleation, growth, and detachment. Moreover, it is inherently multiscale, with processes occurring across macroscopic, microscopic, and molecular scales. A crucial feature of nucleate boiling is the thin liquid film (commonly referred to as a “microlayer”) beneath the bubble, which governs heat and mass transfer.

As shown in Figure 1, this thin film consists of three distinct regions. A nonevaporative thin film, which is found at the center of the microlayer; this region has a film thickness in the nanometre range. Here, the strong interaction between the liquid and the heated surface suppresses evaporation. Some researchers,^1–3^ however, propose that this region contains a dry spot rather than a liquid film. Surrounding the nonevaporative region is an evaporative thin film region, which has a thickness of less than 1 μm. Within this region, the evaporation flux is at its peak, and it accounts for nearly 50% of the total heat transfer,^4^ making it the most critical region for energy exchange. The intrinsic meniscus is an outer region of the thin film, which has an increasing thickness due to capillary forces. The evaporation flux gradually decreases as the film thickness grows, owing to increased conduction resistance. Despite this, the intrinsic meniscus plays a vital role in supplying liquid to the rest of the microlayer and preventing dry-out.

**Figure 1:**
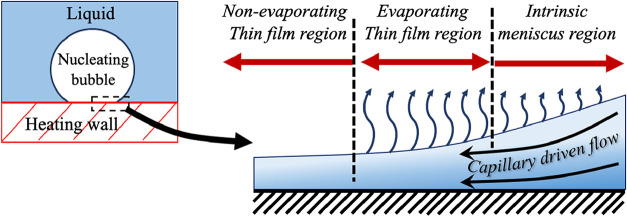
Schematic of nucleate boiling and the thin liquid film below a nucleating bubble.

The nanometre-scale thickness of these films makes them challenging to study using continuum-scale models, where the continuum hypothesis may be invalid. Especially for ultrathin liquid films adjacent to solid walls, strong molecular layering, large density fluctuations, and spatially nonuniform thermodynamic states are observed, which are not captured by continuum-scale models. These effects break the assumptions required for continuum modeling. To address these challenges, molecular-scale simulations have emerged as a powerful tool for investigating the structure and dynamics of these thin films. By capturing atomic-level interactions, molecular dynamics (MD) simulations provide unique insights into the mechanisms driving heat transfer and phase change in nucleate boiling. For example, Long et al.^5^ were among the first to simulate the boiling of argon using molecular dynamics. Wemhoff and Carey^6^ pioneered MD simulations for boiling water, and subsequent studies have primarily focused on understanding the effects of surface structure and wettability on boiling.^7–9^ Ma et al.^10^ have used molecular simulation to study the solid–liquid interfacial resistance during evaporation. Recently, Ozsipahi and Beskok^11^ have studied thin-film evaporation and its mechanisms using molecular simulations, identifying different regions of thin-film evaporation.

In these MD studies, two main types of computational domains are typically used. As shown in Figure 2(a), using a fully filled liquid domain is suitable for observing bubble nucleation during boiling. However, this approach results in pressurization of the domain, as no additional space is available for expansion. This pressurization affects the boiling process because properties such as saturation temperature are pressure-dependent. In the configuration shown in Figure 2(b), in which a vacuum overlies a liquid film or pool, the domain provides space for vapor expansion, accommodating the vapor generated during boiling. However, as the vapor accumulates, its density continually increases, making it difficult to achieve steady-state conditions. Jones et al.^12^ proposed a piston-based system to maintain constant pressure during boiling simulations to overcome these limitations. As shown in Figure 2(c), this method involves placing a piston above the liquid pool and applying a constant force to regulate the pressure over the liquid–vapor system. Using this approach, Shahmardi et al.^13^ studied the effects of nanostructures and wettability on boiling, while Hu et al.^14^ explored pressure-dependent boiling behavior, which cannot be effectively studied using other simulation setups.

**Figure 2:**
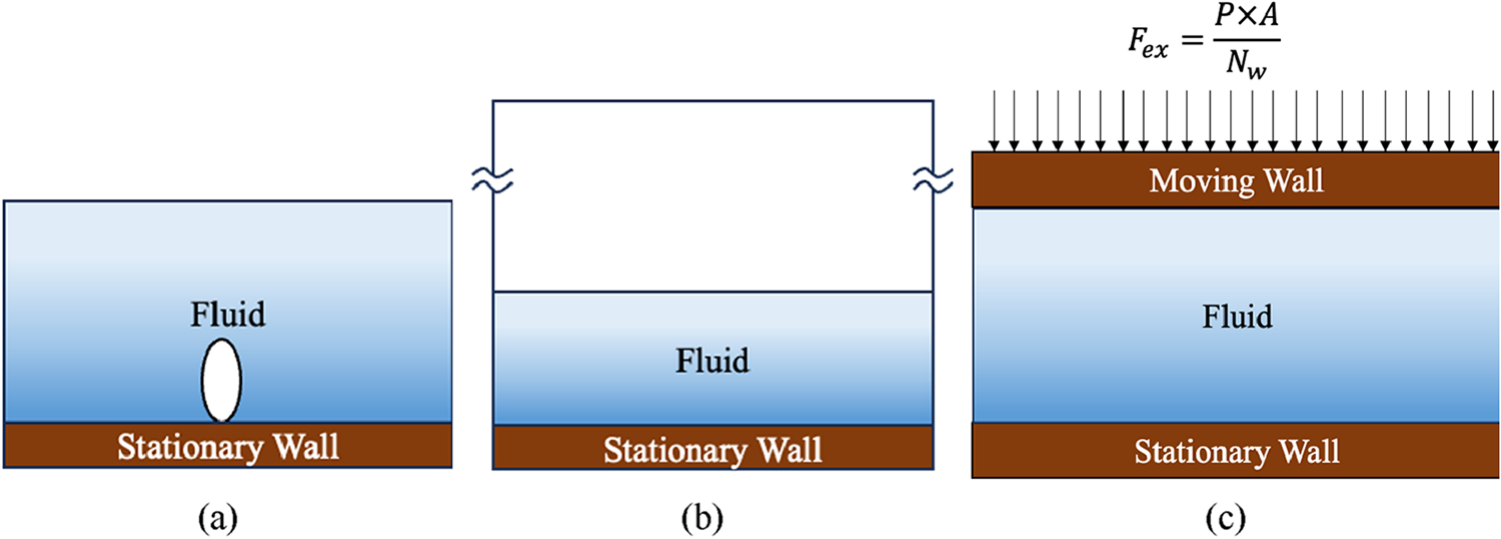
Different types of molecular-scale domains used for simulating boiling (a) liquid packed domain, (b) vacuum overlaid domain (c) piston based isobaric domain.

Despite these advancements, the fundamental mechanisms governing nucleate boiling remain underexplored, and isobaric MD simulations, such as the piston-based approach, hold great potential for addressing these gaps. To the best of our knowledge, no prior molecular dynamics study has evaluated liquid–vapor interfacial thermal resistance under constant-pressure boiling conditions. This is primarily because maintaining a stable isobaric environment during phase change is technically challenging in MD, and no experimental technique currently provides nanoscale, time-resolved interfacial temperature and mass-flux data for direct validation. Thus, the present work offers the first molecular-scale characterization of interfacial resistance under isobaric boiling, filling an important gap between nanoscale physics and continuum-scale boiling models. In this study, we investigate both evaporating and nonevaporating liquid film boiling. The next section presents the numerical model used for the simulations, followed by validation of the model. The subsequent sections detail the results for nonevaporative films and for thick and thin evaporating films. Concluding remarks are provided in the final section.

## Molecular Modeling and Simulation

### Simulation Setup

All the molecular simulations of the present study have been performed by the molecular dynamics (MD) solver of LAMMPS.^15^ Water is utilized as the working fluid, and copper is considered for constructing the heating wall. Water molecules are represented using the SPC/E (Extended Single Point Charge) model, chosen for its high computational efficiency and accurate estimation of water properties across a wide range of conditions. The SPC/E model describes a water molecule as consisting of one oxygen atom and two hydrogen atoms connected by covalent bonds (O–H bonds). The nominal O–H bond length is 1 Å, and the H–O–H bond angle is 109.47°. The charges of oxygen and hydrogen atoms of water are −018476*e* and +0.423*e*, respectively. Here, “*e*” represents the elementary charge, equal to 1.602 × 10^–19^ C. The SHAKE algorithm^16^ is employed to preserve the molecular geometry during simulations.

For the MD simulation, the liquid water film is constructed using face-centered cubic (FCC) units with a lattice constant of 3.45 Å, corresponding to a density of 1.0 g/cm^3^ for liquid water at 1 atm pressure. The copper substrate is also modeled using face-centered cubic (FCC) units. Each FCC unit comprises four copper atoms, with a lattice constant of 3.615 Å, resulting in a density of approximately 8.9 g/cm^3^. This value closely matches the density of copper under standard temperature and pressure conditions. The NVT canonical ensemble has been used in the present study to control the temperature of the fluid and the wall, which performs time integration on the Nose-Hoover thermostatting style.

The nonbonded interactions among water molecules and wall atoms during the MD simulations are effectively modeled using a combination of the Lennard-Jones potential (representing van der Waals forces) and the Coulombic potential (representing electrostatic forces). This combined potential function, given by the following expression, accounts for the contributions from electrostatic, dispersion, and repulsive forces

1 $$U_{LJ}\left(r\right)=4\varepsilon_{\mathrm{ij}}\left[{\left(\frac{\sigma_{\mathrm{ij}}}{r_{\mathrm{ij}}}\right)}^{12}-{\left(\frac{\sigma_{\mathrm{ij}}}{r_{\mathrm{ij}}}\right)}^{6}\right]+\frac{q_{i}q_{j}}{4\pi \varepsilon_{0}r_{\mathrm{ij}}}$$

In this study, *i* and *j* represent two distinct atoms, ε_0_ denotes the permittivity of free space, and ε*_ij_* and σ*_ij_* are collectively referred to as Lennard-Jones (L-J) parameters. Here, a 9 Å cutoff distance has been considered for the L-J potential. Long-range Coulombic interactions are computed using the particle–particle-particle mesh (PPPM) method^17^ with an accuracy of 1 × 10^–4^. In this work, the Lennard-Jones (LJ) interaction between hydrogen and other atoms is neglected because each water molecule is represented by its oxygen atom as per the SPCE water model.^18^ As a result, the interaction between oxygen and other atoms effectively captures the interaction between a copper atom and a water molecule. This simplification significantly reduces computational time without compromising the accuracy of the simulation results.

The copper substrate is constructed by stacking layers of FCC unit cells in all directions, maintaining a consistent center-to-center separation of 3.615 Å between adjacent FCC cells. The entire wall is divided into two distinct layers. In the Fixed Layer, which is the innermost layer, the copper atoms are held fixed without allowing atomic vibrations. This restriction prevents deformation and penetration of the wall, maintains the simulation box volume, and minimizes thermal energy loss from the system. Adjacent to this layer, we have the Phantom Layer, which is thermostated to regulate the temperature of the copper atoms. In this study, one unit cell length is allocated to the fixed layer while the remaining portion of the substrate functions as the phantom layer.

In our MD simulations, the copper substrate is heated to a high temperature. To keep the FCC structure of the copper wall intact, the interactions between solid copper atoms are modeled by introducing artificial harmonic bonds^19^ (in place of metallic bonds). These harmonic bonds create spring-like interactions between Cu–Cu atoms, governed by a spring constant *K*, as follows

2 K=Yd

Here, *Y* is Young’s modulus having a value of 306 GPa, and *d* is the lattice constant. To maintain constant pressure on the fluid, a copper-like piston of 10.845 Å thickness has been placed over the liquid pool. The force *F*_ex_ on individual atoms is calculated from the following equation so that the pressure force is uniformly distributed among all the piston atoms

3 $$F_{\mathrm{ex}}=\frac{P\times A}{N_{w}}$$

where *N*_w_ is the total number of piston atoms.

In case of the nonevaporating film, the piston becomes stationary as the system achieves mechanical equilibrium and maintains the pressure. However, in the case of the evaporating film, due to phase change, the vapor is generated and the system keeps expanding. Hence, under such circumstances, and to accommodate the extra volume, the piston rises to keep the pressure constant, until the supply of liquid has been exhausted. In the present study, to measure the rate of evaporation, we kept enough liquid in the domain so that it is never exhausted before the end of the simulation. We also measure the evaporation rate under such a nonequilibrium state.

The interaction between this piston wall and the water molecules is crucial for the boiling simulations. In previous studies,^12,13,13^ researchers have used heating wall atoms (i.e., copper atoms in the present study) for the piston as well. However, these walls often impart strong disjoining pressure over the fluid, which alters the boiling characteristics of the fluid. Here, we eliminate this effect by increasing the length scale of the L-J potential so that the force field is stretched and its attractive part is beyond the cutoff radius. This is done by setting σ equal to the cutoff radius (9 Å), and as a result, we ended up with a repulsive-only force field.

It is important to note that this piston wall is not intended to represent a physical solid surface; rather, it serves as a computational device to impose the desired pressure, analogous to the presence of a long vapor column in an actual system. Therefore, it should not introduce additional disjoining pressure or modify the liquid structure. The bottom heating wall retains standard LJ interactions and thus provides the physically relevant disjoining pressure, while the repulsive-only piston wall prevents artificial fluid–solid adhesion, ensuring that pressure control does not interfere with the boiling dynamics.

### Validation

To validate the system’s capability to maintain constant pressure, argon was used as the working fluid. Figure 3(a) illustrates the overall simulation domain. The piston area was set to 20 nm × 20 nm, with the thickness of the argon pool defined as 25 nm. A piston was positioned above the gas atoms, and a constant force was applied uniformly across the piston to maintain a pressure of 1 atm. No bottom wall was implemented; instead, the bottom boundary was defined as reflective to prevent the loss of atoms. All side boundaries were set as periodic. The argon was equilibrated at −180 °C, slightly above its saturation temperature. The resulting pressure and density profiles are shown in Figure 3(b). Both profiles exhibit minimal fluctuations, with the average pressure (0.99 atm) and density (5.28 g/cm^3^) being within 1% of their expected values.^20^ This confirms the system’s ability to effectively maintain constant pressure.

**Figure 3:**
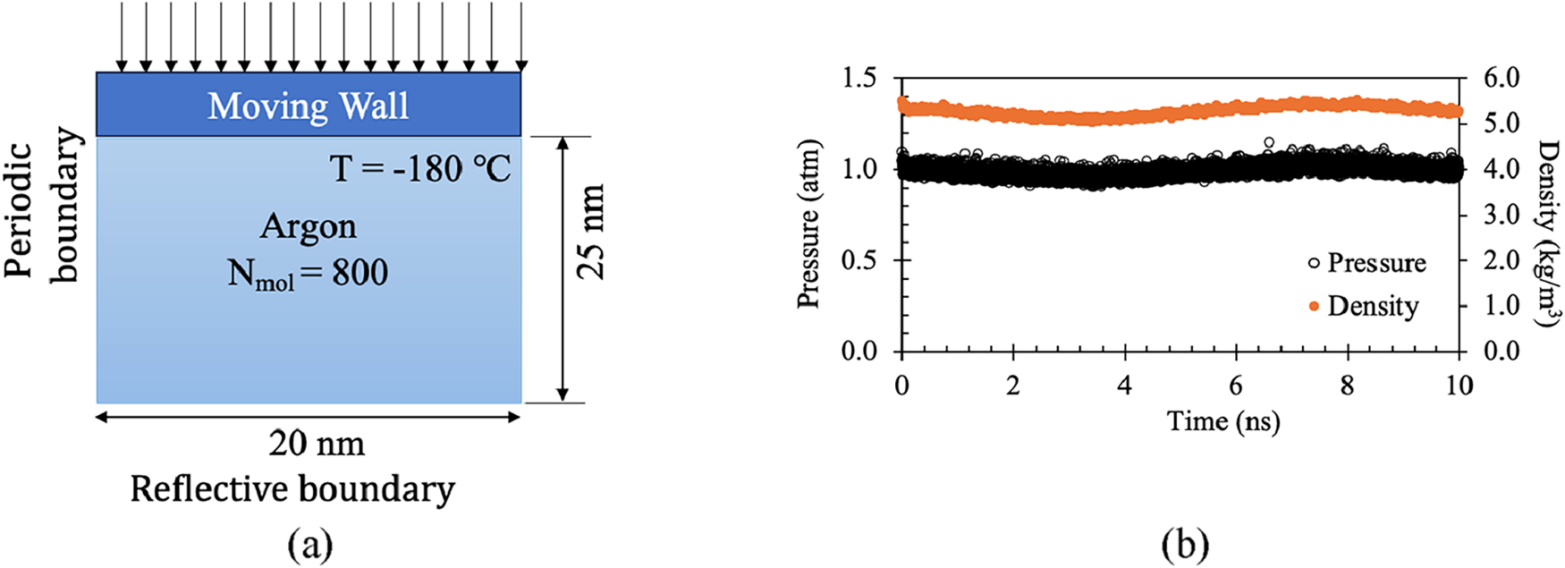
Validation of the system with argon gas (a) domain (b) variation of pressure and density.

Furthermore, we have tested our setup with water to assess its capability to cope with different conditions. A bottom wall was added beneath the fluid pool this time, as shown in Figure 2(c). The thickness of the plate is kept the same as that of the moving piston. Here, the dimension of the steam block is 18.07 × 18.07 × 149.6 nm^3^, and the force on the piston is adjusted to maintain the system’s pressure at 1 bar. It has been observed that because of the repulsive-only force field between the piston and fluid, the piston often oscillates near the fluid for a long time, especially when water is used as a working fluid. This unnecessarily increases the equilibration time and computational time. To avoid this, a damping force (*F*_d_) is added to the piston, which is proportional to the piston speed (*V*_p_)­

4 $$F_{d}=-\zeta V_{p}$$

Our results suggest that a value of ζ = 0.1 provides adequate damping and rapid equilibration for the present domain (Figure S1).

As shown in Figure 4(a), our MD predictions lead to a steam equilibrated at 125 °C with an approximately constant density; the average pressure also appears to be essentially constant at this temperature. To understand the system’s dynamic response, the steam temperature is gradually increased from 125 to 225 °C over 2 ns. As a result, we can see in Figure 4(b) that the pressure rises to 1.4 atm in the initial 2 ns time before reaching a steady value; the density also decreases to a plateau value. In Table 1, the equilibrated density and pressure values are shown; for both cases, the errors are less than 20%.

**Table 1:** Average Equilibrated Pressure and Density of Water at Different Temperatures

		pressure (atm)			density (kg/m^3^)		
	temperature (°C)	standard		result		% error	
liquid	25	1.0	997	-	989	-	0.8
	95	1.0	962	-	940	-	2.3
vapor	125	1.0	0.550	1.05	0.623	5	13.3
	225	1.0	0.437	1.16	0.521	16	19.2

**Figure 4:**
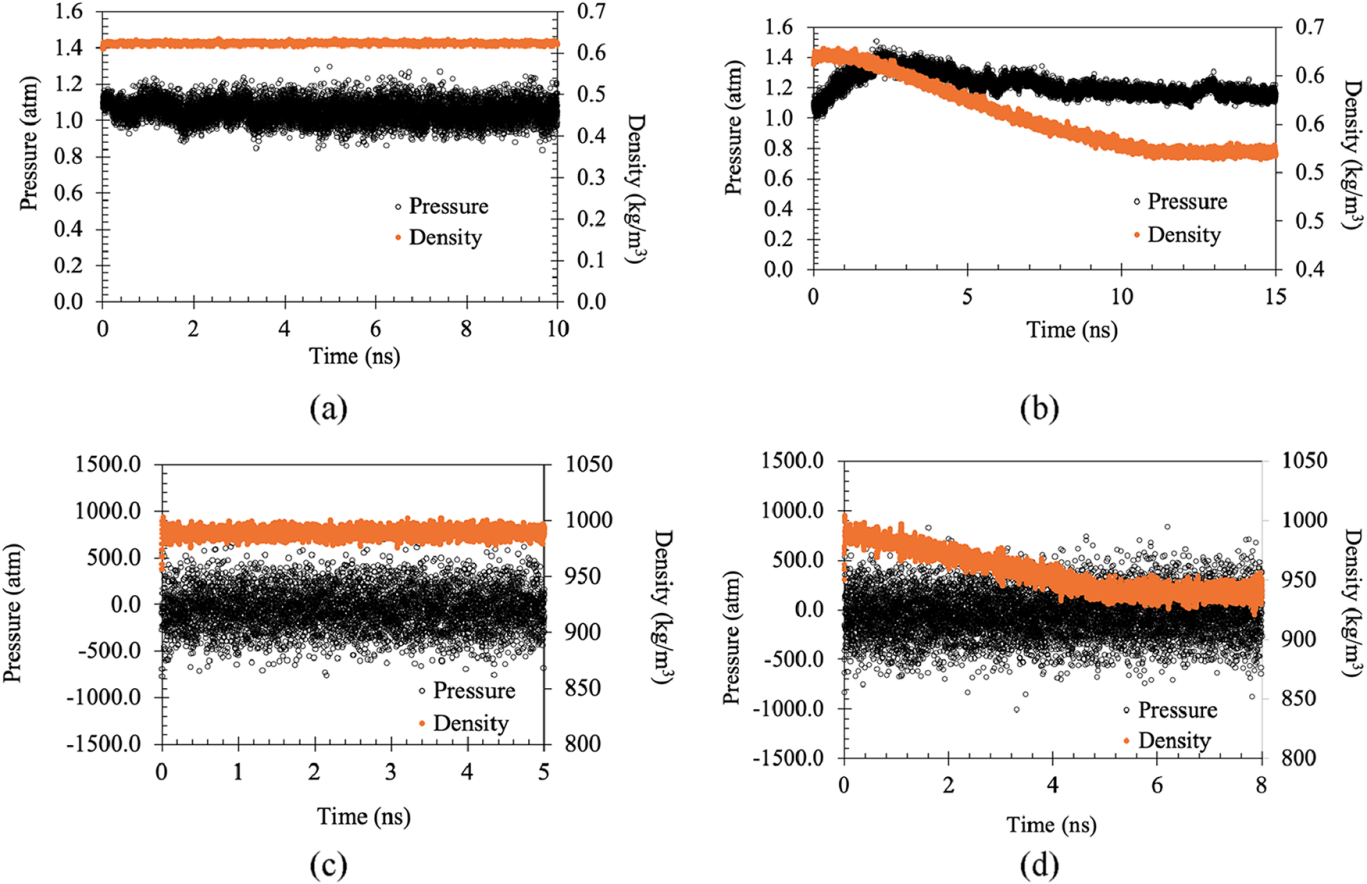
Variation of water density and pressure at different temperatures and states (a) steam at 125 °C, (b) steam at 225 °C, (c) liquid at 25 °C, and (d) liquid at 95 °C.

A similar simulation has also been performed with liquid water, which, due to its higher density, requires a much smaller domain than the simulations involving steam discussed above; here, the dimensions of the liquid block are 2.53 × 2.53 × 15.6 nm^3^ and the domain is equilibrated at 25 °C. As shown in Figure 4(c), the liquid pressure fluctuation is much higher (±500 atm) than in the case of steam because of the finite size of the domain. Following an increase in the water temperature from 25 °C to 95 °C over 5 ns, the density appears to decrease before saturating to a constant value, as shown in Figure 4(d). The average density of both of these cases shows a low deviation from the expected value (see Table 1).

No such deviation was observed in the case of argon, which does not involve a stationary wall. This indicates that the presence of the wall contributes to the observed deviation in the water simulations. Moreover, argon, being a noble gas with a simple atomic structure, exhibits very small fluctuations; therefore, no piston damping was required for its equilibration. In contrast, for water, a damping coefficient was introduced to accelerate equilibration, which had a slight influence on the pressure values and resulted in a minor deviation. Nevertheless, the overall deviation remains within an acceptable range.

Furthermore, to examine the capability of the system to simulate a phase change scenario at different pressures, we have heated the water to different temperatures. Here, the same domain has been utilized where a liquid water block has been placed between the static and moving walls. Initially, the pressure is kept constant at 1 atm, and the piston movement is studied, as shown in Figure 5(a). Here, one can see that the piston is essentially stationary at 90 °C, but as the temperature crosses the saturation temperature 100 °C mark, the piston rises monotonically. Moreover, as the temperature rises further, the piston moves faster. This piston speed is dependent on the rate of evaporation. After some time, the piston speed is expected to saturate when the evaporation reaches its equilibrium value. This equilibrium rate of evaporation is dependent on different conditions, e.g., degree of superheat, pressure, liquid film thickness, surface structure and many more. A few of those aspects will be discussed in the subsequent sections. Similar simulations were repeated with a pressure of 10 atm. Here, even at 130 °C, the piston remains stationary. At 175 °C, the piston shows small oscillations as it nears the saturation temperature of 180 °C. At 200 °C, the piston starts moving up monotonically, showing a clear indication of boiling. Hence, our MD simulations within the present isobaric system provide predictions which agree with published saturation temperatures at various pressures.^21^

**Figure 5:**
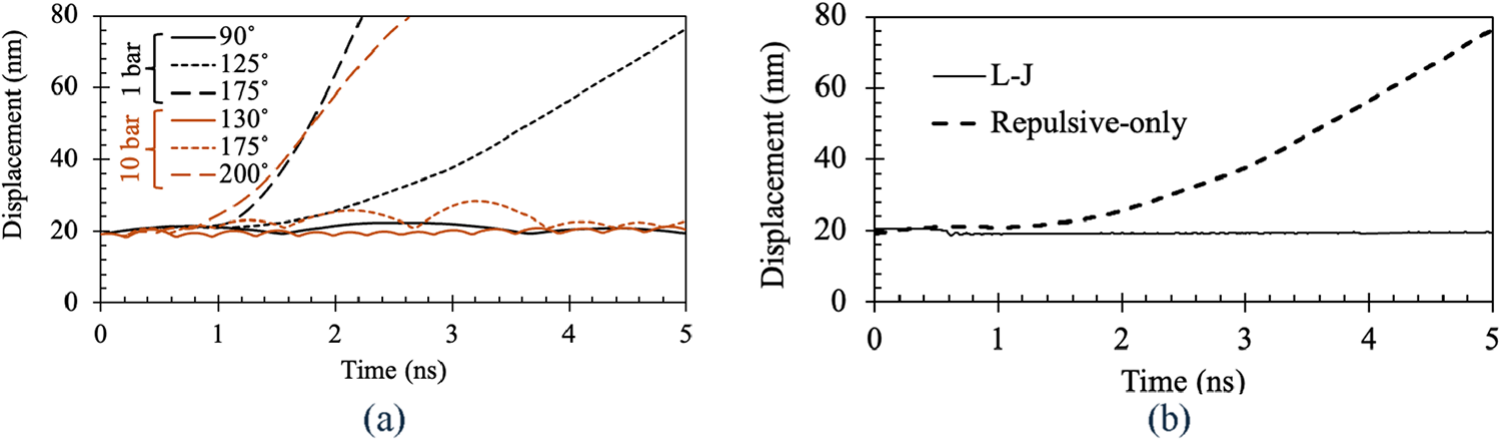
(a) Piston displacement for water at different temperatures and pressures 1 and 10 atm, and (b) comparison of L-J and repulsive-only force field simulation at 125 °C and 1 atm pressure.

We also performed two simulations at 1 atm pressure and 125 °C with a repulsive force field in one simulation and an L-J force field in the other. In Figure 5(b), a comparison of the piston movements associated with these two simulations is shown. Here, one can see that for the L-J force field, the piston does not move at all, but for the repulsive-only force field, the piston rises monotonically, showing clear evidence of evaporation. Hence, the L-J force field hinders the boiling even above the saturation temperature, which justifies the use of the repulsive-only force field in our simulations.

## Results

In this section, we discuss the evaporation dynamics of three types of films: (a) nonevaporative films, (b) evaporating thick films, and (c) evaporating thin films. Figure 6 illustrates the simulation domains corresponding to each case. For the thick evaporating film, the stationary wall is omitted because only the interfacial region is simulated. In contrast, a stationary wall is included in the other two cases, as wall–fluid interactions play a key role in the boiling dynamics. Additional details for each configuration are provided alongside the respective results.

**Figure 6:**
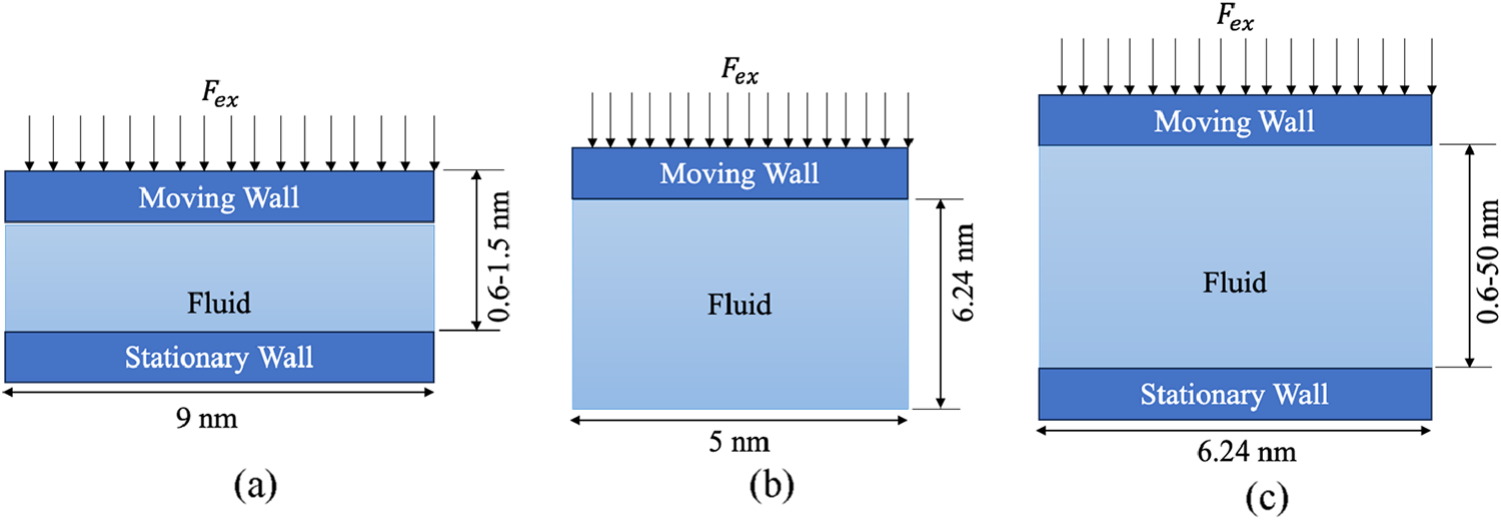
Domain size and description for different cases: (a) Nonevaporative film, (b) Evaporating thick film and (c) Evaporating thin film.

Selecting appropriate domain dimensions is critical in molecular dynamics simulations, where a minimum number of molecules is required to obtain statistically meaningful thermodynamic and transport properties. Following literature recommendations^22^ (typically involving ≥1000 molecules), the simulation domains were designed to strike a balance between physical fidelity and computational feasibility. For the nonevaporative thin film, achieving the smallest possible film thickness while maintaining sufficient molecules required a lateral width of 9.048 nm, yielding 1682 water molecules in the thinnest configuration. For the thick evaporating film, the block height was not constrained, allowing the use of a 4.992 nm cubic domain containing 4092 molecules. The thin evaporating film posed the greatest challenge, as its thickness varies from 0.6 to 50 nm. To maintain consistent lateral dimensions across this range, a width of 6.24 nm was selected, resulting in approximately 1200 molecules for the thinnest film and up to 64,000 molecules for the thickest. This approach ensures adequate statistical sampling while avoiding prohibitively high computational cost.

### Nonevaporative Film

The nonevaporative film thickness corresponds to the maximum film thickness beyond which evaporation starts. Hence, to detect the film thickness, we need to heat the water film with different thicknesses. The disjoining pressure imparted by the wall on the liquid film is the primary reason behind the nonevaporative film. Hence, it is highly dependent on the wall–fluid interaction and the wettability of the wall. Initially, we started with ε = 0.3 the water and the bottom wall particles. This potential corresponds to a contact angle of 81.9°. With this interaction potential, we could not find a stable nonevaporative film under any pressure.

Next, for ε = 1.452, which corresponds to a contact angle of 18.5̊, we kept the pressure over the liquid film constant at 1 atm with the help of the piston, and heated the film and walls to 130 °C. As expected, the thicker film begins to boil, and the piston rises, as shown in Figure 7(a) in terms of normalized piston height, which has been nondimensionalised over initial height. Here, the piston height is measured from the lower end of the stationary wall, i.e., the lowest point of the domain. However, for the thinnest film (0.62 mg/m^2^), the piston does not move much, and it stabilizes at a particular height. Similarly, in Figure 7(b), the piston movement for 10 atm is shown. Here, we can see that for the 0.62 mg/m^2^ and 0.92 mg/m^2^ film thickness, the piston hardly moves. Hence, the film thickness corresponding to 0.92 mg/m^2^ can be thought of as the nonevaporative film thickness at 10 atm. Similarly, the thickness of nonevaporative films has been detected for other pressure values.

**Figure 7:**
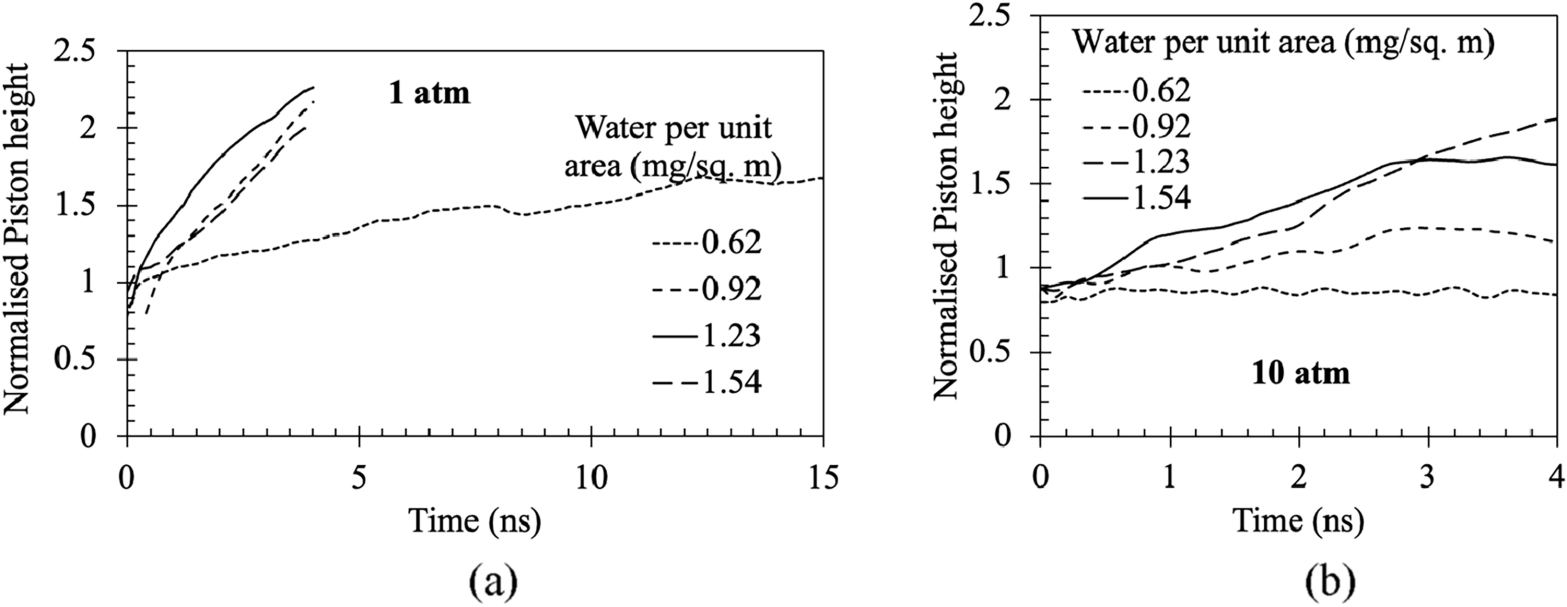
Piston movement for different film thicknesses at (a) 1 atm and (b) 10 atm. Here, ε = 1.452, which corresponds to a contact angle of 18.5° and the film and wall are heated to 130 °C.

Figure 8(a) presents a rendering of the nonevaporative film obtained from the MD simulations. Here, we can clearly see that the film thickness increases with pressure, and the vapor density above the film also increases. As the vapor density increases, the vapor molecules are more prone to returning to the film, and it becomes easier for the film to accommodate an increasing number of molecules. As MD is a particle solver, it is essential to convert the particle to volume when calculating the film thickness. Here, a Gaussian kernel point-to-volume interpolator^23^ has been used for this purpose, and a kernel footprint of 3Å (as water molecule diameter is approximately 3Å; N.B. for SPCE water molecule σ = 3.165 Å) has been considered for this calculation. In Figure 8(b), the change in film thickness with pressure can be observed clearly. At lower pressure, the thickness change is more prominent; beyond 10 bar, the film thickness changes very little. At 20 atm, the maximum film thickness is observed; beyond that, the thickness reduces slightly. The error bars in Figure 8(b) reflect the uncertainty associated with the discrete thickness increments used to identify the nonevaporating film limit.

**Figure 8:**
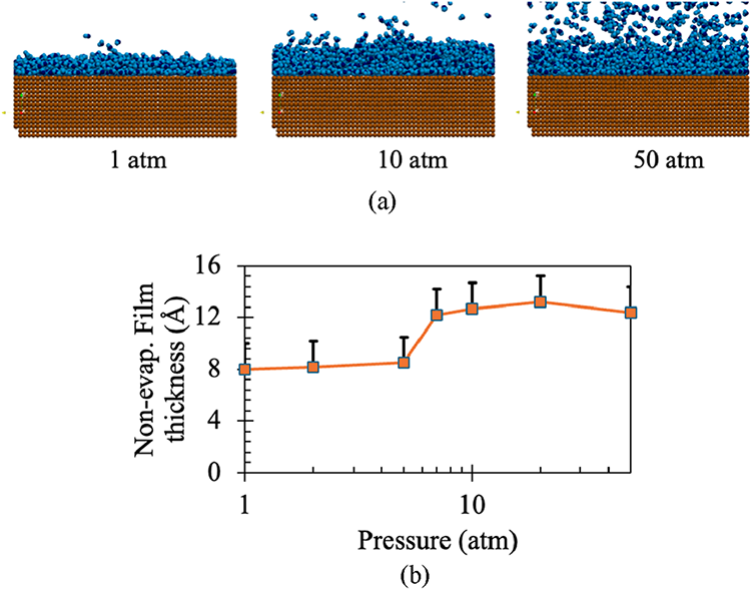
(a) A rendering of the nonevaporative film thickness obtained from the MD simulations at different pressures, (b) Variation of nonevaporative film thickness with pressure. Here, ε = 1.452, which corresponds to a contact angle of 18.5° and the film and wall are heated to 130 °C.

### Thick Evaporating Film

Evaporation through a thin liquid film involves the transfer of heat from the substrate to the liquid–vapor interface, where it is consumed during phase change. The resulting evaporation heat flux is governed by two mechanisms: heat conduction through the liquid layer and the molecular energy exchange occurring at the interface. These contributions can be described as thermal resistances in series—the conduction resistance of the liquid film and the interfacial thermal resistance. The conduction resistance depends on the film thickness (δ) and the thermal conductivity of the liquid (*k*_*l*_), while the interfacial resistance arises from the steep gradients in thermophysical properties across the interface. The overall heat flux (q_l_^″^) can therefore be written as

5 $${q_{l}}^{\prime \prime }=\frac{\Delta T}{\delta /k_{l}+R^{i}}$$

From a mass-transfer perspective, the evaporation heat flux is also related to the temporal variation of film thickness by

6 $${q_{l}}^{\prime \prime }=-L\rho_{l}\frac{\partial \delta }{\partial t}$$

Here L is the latent heat of evaporation, ρ_*l*_ the liquid density, and $\frac{\partial \delta }{\partial t}$ is the rate of film depletion. When only the interfacial region is considered, the conduction resistance may be neglected. This simplification is appropriate at the nanoscale because the interfacial resistance becomes much larger than the bulk conduction term. For reference, the ideal interfacial resistance of water at 1 atm corresponds to the conduction resistance of a water film approximately 40 nm thick. Since the films in our simulations are significantly thinner than this threshold, the interfacial resistance dominates the overall heat transfer. Neglecting the conduction term, therefore, yields

7 $$R^{i}=-\frac{\Delta T}{L\rho_{l}\frac{\partial \delta }{\partial t}}$$

To ensure that conduction resistance does not influence the results, the entire water film is equilibrated at a uniform temperature before the evaporation process begins.

Tecchio et al.^24^ tried to measure the film depletion rate experimentally to calculate the variation of the resistance over the bubble life cycle. Thus, if the rate of film depletion is measured from the MD simulations, then *R* ^*i*^ can be calculated accurately.

As observed in the previous section, the wall strongly influences the film evaporation dynamics. Hence, it is very important to ensure that the solid wall does not affect the evaporating interface. Ideally, to achieve this, we need to simulate a microfilm, which is computationally too expensive. Alternatively, it would be better if the bottom wall could be removed. However, removing the bottom wall is not sufficient because that will create another liquid interface near the bottom boundary, and some water molecules might evaporate from there. Therefore, we need to apply an extra force on the molecules near the bottom boundary, which will make them feel as if they are inside a liquid pool, not at the interface.

For example, in Figure 9(a), a schematically bottommost layer of the liquid layer has been shown. Here, a representative molecule is shown at a distance of *h* from the bottom boundary. As *h* is smaller than the cutoff radius (*r*_cut_) of the interaction force field, its cutoff radius extends out of the domain. We therefore need to add an additional force to this molecule to represent the force from the out-of-domain molecules that lie within the cutoff radius. Zhou et al.^25^ have calculated a similar force field for gases from molecular simulations. However, here we will try to derive this force field analytically by dividing a spherical cap into infinitesimally thin caps of *dr* thickness and radius *r*, as shown in Figure 9(a). Hence, the potential (*E*_b_) due to this spherical cap will be

8 $$E_{b}\left(h\right)=\sum {E_{b}}_{i}=\int_{h}^{r_{\mathrm{cut}}}\rho_{n}E_{\mathrm{lj}}\left(r\right)dV$$

The local number density (ρ_*n*_) of the molecules can be described in terms of radial distribution function (*g*(*r*)) and average number density ( $\overset{®}{\rho_{n}}$ ) as follows

9 $$\rho_{n}=\overset{®}{\rho_{n}}g\left(r\right)$$

Here, *g*(*r*) can be statistically calculated from any molecular dynamics simulation of water. Replacing ρ_*n*_, the following expression can be expressed in terms of *g*(*r*) replacing *dV* geometrically in eq 8

10 $$E_{b}\left(h\right)=2\pi \overset{®}{\rho_{n}}\int_{h}^{r_{\mathrm{cut}}}g\left(r\right)E_{\mathrm{lj}}\left(r\right)r\left(r-h\right)dr$$

Finally, the above equation is solved numerically for different values of *h*. The resulting potential and force field per unit mass are shown in Figure 9(b). This potential and force field helps to remove any density layering, and a smooth, uniform density profile is obtained as shown in Figure 9(c).

**Figure 9:**
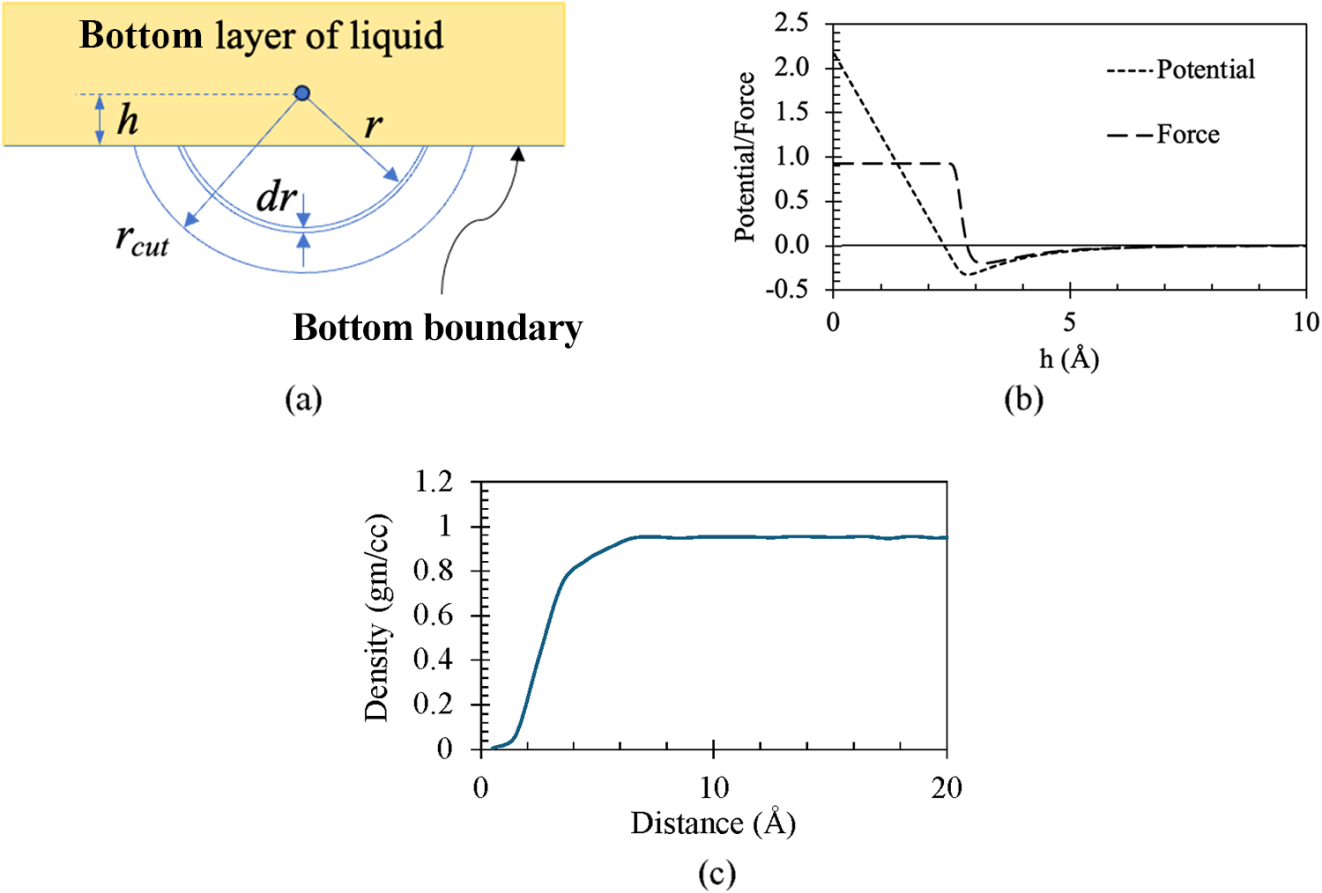
(a) Schematic of boundary force, (b) Boundary potential (and associated force) for water obtained from the solution of eq 10, (c) Resulting density profile upon applying the boundary force shown in panel (b).

Using this potential and force field, simulations were performed by heating the liquid pool at different temperatures and 1 atm pressure. From the results obtained, the piston movement and the film depletion are tracked. Here, piston height has been measured from the lowest point of the liquid film, i.e., the lower boundary of the domain. In Figure 10(a), the results of boiling at 130 °C are plotted, showing low-speed piston displacement during the early stages of the dynamics, which, as boiling commences, gradually accelerates. After some time (∼7 ns), the piston speed saturates at ∼300 m/s. As shown in Figure 10(b), initially, a small increase in film thickness is observed because of the expansion of the liquid film due to heating. Thereafter, the film depletion rate begins to rise, and after 10 ns, it appears to saturate. The ratio of piston speed to film depletion rate is approximately 1600, which is quite close to the ratio of water-steam density at 1 atm. This verifies that the pressure and the densities are maintained quite well throughout the simulation.

**Figure 10:**
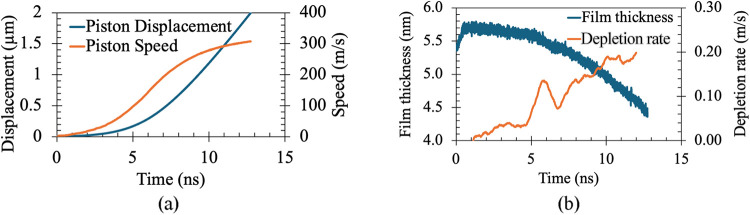
Temporal variation of (a) piston displacement and speed, (b) Film thickness and the rate of depletion for boiling at 130 °C

Similarly, the film thickness has been tracked for other temperatures up to 40 °C superheat until saturation is achieved. Once the film thickness is saturated, the slope is calculated with block-averaged regression. It has been found that a block size of 200 time steps (i.e., 0.2 ns) is most suitable for the data set. The corresponding standard error has also been calculated and reported in Table 2.

**Table 2:** Interfacial Thermal Resistance and Accommodation Coefficient of Water at 1 Bar

temp	120 °C	130 °C	140 °C
$\frac{\partial \delta }{\partial t}$ (m/s)	0.116 ± 0.007	0.184 ± 0.005	0.257 ± 0.015
*R* ^*i*^ (μK/W)	0.079 ± 0.005	0.076 ± 0.003	0.072 ± 0.005
F	0.89 ± 0.03	0.9 ± 0.017	0.93 ± 0.035

Further, the interfacial thermal resistance was calculated from eq 7. Theoretically, the interfacial thermal resistance is calculated from the following equation

11 $${R^{i}}_{\mathrm{theo}}=\frac{T_{\mathrm{sat}}\sqrt{2\pi R_{g}T_{\mathrm{sat}}}}{2L^{2}\rho_{v}}$$

For water at 1 atm, the value of this theoretical resistance is 0.063 μK/W. To account for deviations from the ideal kinetic-theory prediction of interfacial phase change, we introduce the accommodation coefficient F following the Scharge model.^26^ The coefficient F represents the fraction of interfacial molecules that actually evaporate (or condense), relative to the maximum rate predicted by kinetic theory. In the Schrage formulation, the net mass flux is reduced by the factor (2−F)/(2F) , which arises from balancing the outgoing evaporation flux with the incoming condensation flux when only a fraction F of molecules participate in phase change. Incorporating this effect leads to the following theoretical expression for the interfacial thermal resistance

12 $$R^{i}=\frac{\left(2-F\right)}{2F}\frac{T_{\mathrm{sat}}\sqrt{2\pi R_{g}T_{\mathrm{sat}}}}{L^{2}\rho_{v}}$$

Comparing eqs 11 and 12, the accommodation coefficient can be calculated as

13 $$F=2/\left(1+\frac{R^{i}}{{R^{i}}_{\mathrm{theo}}}\right)$$

In this way, using eqs 7 and 13, the interfacial thermal resistance and accommodation coefficient are calculated from the simulation and tabulated in Table 2. Here, we can see that the rate of film depletion increases with temperature, but the resistance value does not vary appreciably. As a result, the accommodation coefficient is around 0.9.

### Thin Evaporating Film

In the previous two subsections, we have dealt with the evaporation of the thickest and thinnest films. Here, we try to shed some light on the evaporation of intermediate-thickness nanofilms. For that, we repeated the same procedure as for the thick evaporating film, but in the presence of a solid wall. We have varied the film thickness up to 50 nm and kept the temperature of the water fixed at 130 °C for all the simulations. Like the thick evaporating film, we continued the simulation until the piston speed and film depletion rate reached a steady value. In Figure 11(a), the film depletion rate has been plotted against time for a few representative thicknesses. Similar to the thick-film case, the block-averaged slope was used to compute the film depletion rate for the thin film, and the 95% confidence interval was found to be within 20% of the mean value. The simulations have been repeated for two different wettabilities using ε = 0.3 and ε = 1.452.

**Figure 11:**
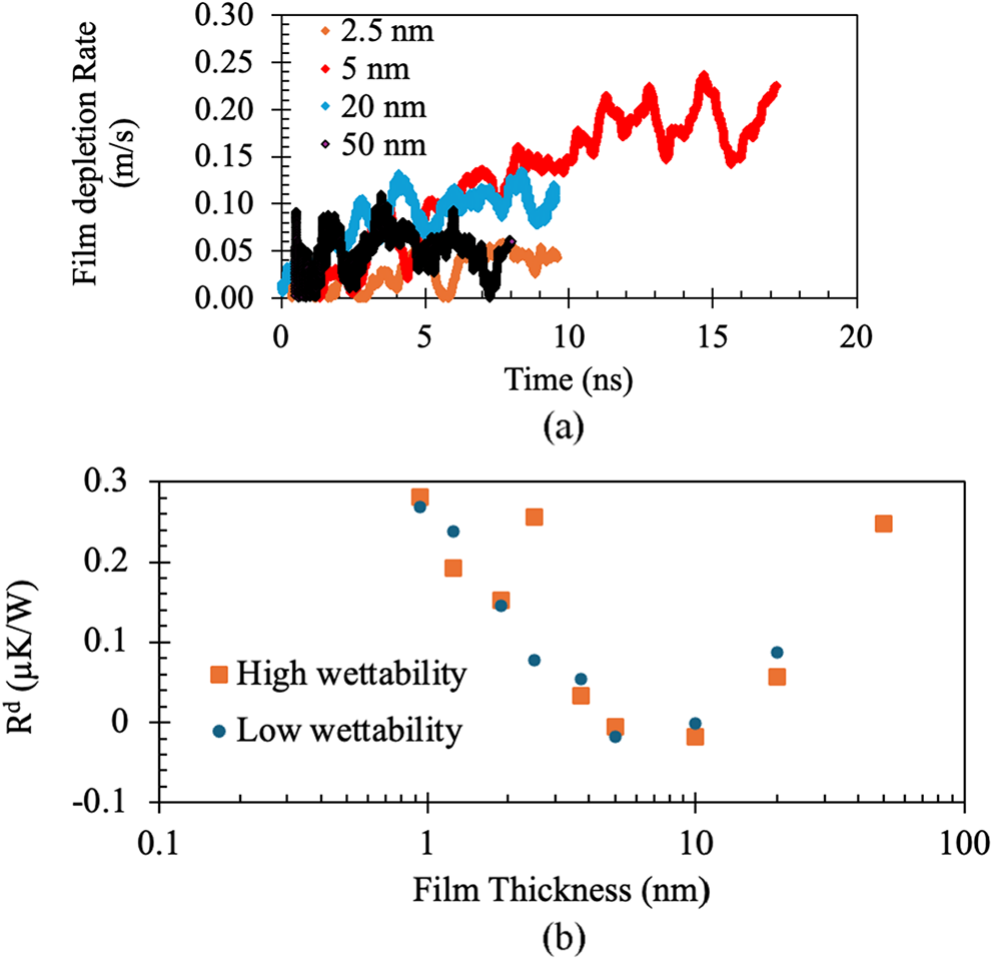
(a) Variation of film depletion rate with time for different film thickness (b) Variation of additional resistance a with film thickness for two different wettability.

From Figure 11(a), it is evident that the film depletion rate in the presence of the solid wall is substantially lower than the corresponding vapor removal rate observed in Figure 10(b), where no wall is present. This clearly demonstrates that the solid wall imposes an additional resistance to phase change, arising from the disjoining pressure.

Including this resistance as *R* ^*d*^ in eq 5, the overall heat flux becomes

14 $${q_{l}}^{\prime \prime }=\frac{\Delta T}{\delta /k_{l}+R^{i}+R^{d}}$$

Combining this with the mass-transfer expression (eq 6) and neglecting conduction resistance in the interfacial zone yields

15 $$R^{d}=-\frac{\Delta T}{L\rho_{l}\frac{\partial \delta }{\partial t}}-R^{i}$$

Since *R* ^*i*^ is already known from our earlier study, the disjoining thermal resistance *R* ^*d*^ can be directly obtained using the simulated film depletion rate.

Figure 11(b) shows the variation of the disjoining thermal resistance with film thickness for both wettability conditions. As expected, the resistance is very high for extremely thin films under high-wettability conditions, corresponding to the nonevaporative layer. Surprisingly, after the initial decay, the trend is nearly identical for both wettabilities and *R* ^*d*^ reaches a local minimum at approximately 5–10 nm before increasing again at larger thicknesses.

These trends can be rationalized by considering that, close to the solid surface, strong liquid–solid interactions generate a significant disjoining pressure that the interfacial molecules must overcome before evaporation can occur. This results in a large resistance for ultrathin films. As the film thickens, the wall–fluid influence weakens, and the disjoining resistance decreases. Beyond a certain thickness, however, density fluctuations begin to reintroduce resistance, leading to the observed rise in *R* ^*d*^.

In Figure 12, we plot the density fluctuation near the interface for various film thickness values; for comparison, the case in which no wall is present is also shown. The fluctuations are obtained by first dividing the domain into 1 Å layers and calculating the average density for each over a time period of 0.01 ns. Then, the standard deviation of the density is calculated for each layer. It is seen clearly that the amplitude of the fluctuations is particularly high near the interface for large film thicknesses. This is due to the effect of the wall underlying the film, which acts to suppress the fluctuations; in the bulk, the fluctuation amplitude is virtually independent of film thickness. The stabilizing effect of the wall is corroborated by inspection of the no-wall case, in which the fluctuation amplitude increases even in the bulk in comparison to the other cases shown in Figure 12.

**Figure 12:**
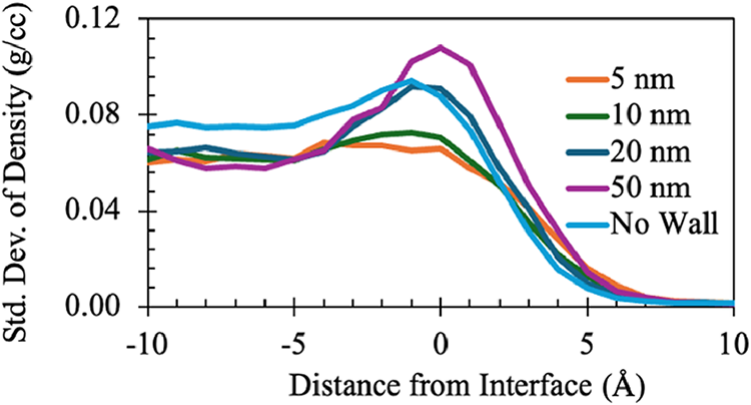
Estimate of density fluctuation near the interface for different film thicknesses

The density fluctuation has a strong effect on evaporation. In the previous section, we have seen that at 130 °C the piston moves at a velocity of 300 m/s, which is effectively the drift velocity of the steam from the liquid interface. For the high fluctuation amplitudes observed at large film thicknesses, this drift velocity will also vary and may even approach the sonic velocity (∼500 m/s at 130 °C and 1 atm pressure). At such high velocities, compressibility effects become appreciable, and the density of the vapor phase close to the interface rises, which has an adverse effect on evaporation. This is because on the vapor side of the interface, there will be more vapor molecules available to condense back to the liquid phase. This ultimately results in an increase in resistance for the high film thickness values examined in the present work.

A solid wall not only alters the density fluctuation, but it also changes the average bulk density. We have calculated the bulk densities for different film thicknesses. While calculating this bulk density, we have chopped off 1 nm of the top layer to get rid of the density change due to the interface. Also, we have chopped off 1 nm of the bottom layer to get rid of the layering of water molecules near the wall. The rest of the film is considered for bulk density calculation. For different film thicknesses, the bulk densities came around 0.93 g/cm^3^. However, for the liquid film without a wall, the bulk density was 0.95 g/cm^3^. That shows that for smaller scale (<50 nm), the bulk density does not show much change; however, in larger scale, we can expect a significant change in bulk density. This rise in average bulk density will enhance the availability of molecules for evaporation at the interface, ultimately reducing the resistance.

As density fluctuations have a strong effect on the overall boiling phenomenon, it would be beneficial to investigate them further. The Vibrational Density of States (VDOS) is a popular concept in molecular dynamics for understanding the nature of vibrations. This can be calculated from the velocity autocorrelation function (VACF *C*_*vv*_) as follows

16 $$C_{\mathrm{vv}}\left(t\right)=\frac{1}{N}\sum_{i=1}^{N}\frac{\left\langle v_{i}\left(0\right).v_{i}\left(t\right)\right\rangle }{\left\langle {v_{i}\left(t\right)}^{2}\right\rangle }$$

17 $$\mathrm{VDOS}\;\left(\omega \right)=\int_{0}^{\infty }C_{\mathrm{vv}}\left(t\right)e^{-i\omega t}dt$$

For this calculation, we can consider velocity data samples of 0.05 ns at an interval of 1 ps from 4000 sample particles. Only O atoms have been considered for this calculation, as the O atom represents the molecular center of mass. It is true that including the H atom would have provided a better picture of the higher frequency vibrations generated from the OH bond vibrations. However, for heat transfer, lower-frequency vibrations are more important, so we have neglected them to reduce computational cost.

In Figure 13(a), the VDOS of vapor and liquid water is presented. Here, one can see that lower-frequency vibrations (<10 THz) are more dominant in the case of vapor compared to liquid water. Interfacial resistance is highly dependent on these frequencies. The lower the mismatch of VDOS between the phases, the lower will be the interfacial resistance. Hence, clearly, if the VDOS of liquid water can be increased somehow at lower frequencies, it will result in a reduction in interfacial resistance.

**Figure 13:**
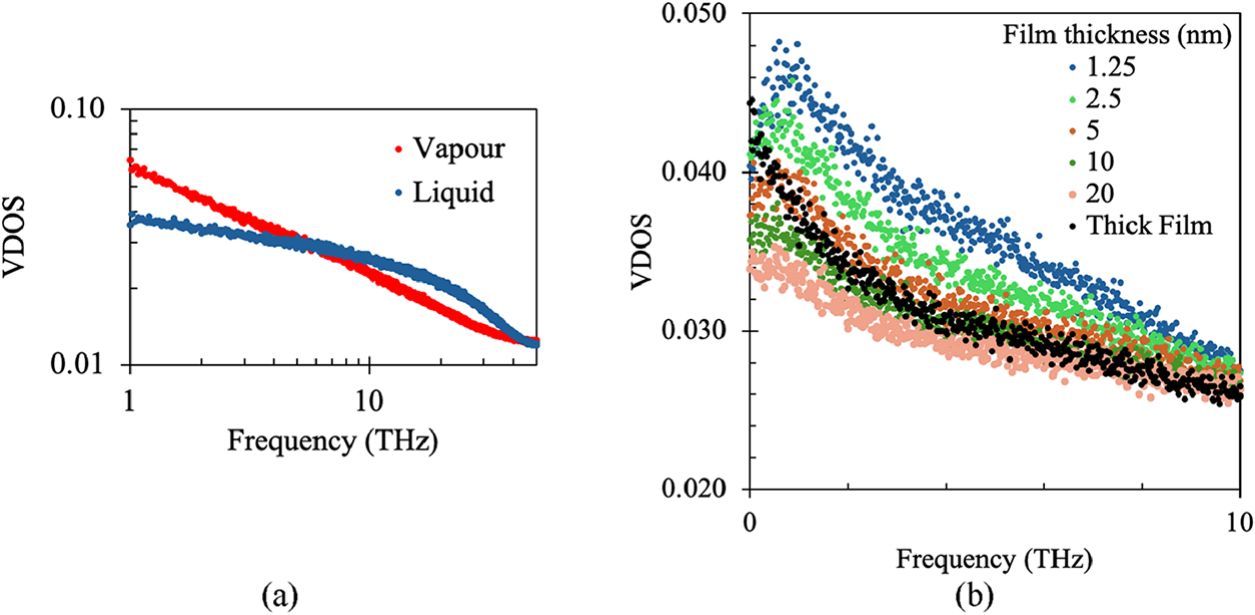
(a) Comparison of VDOS between vapor and liquid water and (b) Comparison of VDOS for different thicknesses of liquid water film.

In Figure 13(b), a comparison of VDOS among liquid films with different thicknesses has been plotted. Here, thick film refers to the film without any walls (as discussed in the previous section). In the case of thick film, we cannot see any maxima point. VDOS reduces in frequency. However, for all finite-thickness films, a peak can be observed around 1 THz. This peak is more prominent in the case of a thinner liquid film. Hence, this shift of VDOS is induced on the liquid film by the solid surface. More significantly, with the increase in film thickness, the VDOS decreases for these lower-frequency zones. That means that at lower thickness, liquid VDOS shows a lower mismatch with the vapor VDOS, and this mismatch increases with an increase in thickness. This increased mismatch of VDOS would try to increase the interfacial resistance. This supports our finding that resistance increases with thickness, particularly when the thickness exceeds 10 nm. However, at a liquid film thickness of less than 5 nm, the disjoining pressure is too strong, so we do not see a further reduction in interfacial resistance upon decreasing the film thickness.

While the existence of a minimum in the additional disjoining resistance is a robust physical feature observed in the present simulations its precise location is expected to depend on the solid–liquid interaction characteristics, including the wall material and force-field representation, which influence density fluctuations and vibrational coupling at the interface, suggesting that systematic variation of the surface material and interaction models could further elucidate this behavior.

## Conclusion

This study used piston-based isobaric molecular simulations to investigate thin, nonevaporative, and thick evaporating films. Both the Lennard-Jones (LJ) and repulsive-only force fields were employed to model the interaction between the piston and the working fluid. It was observed that the LJ force field suppressed boiling due to the additional disjoining pressure it introduced. In contrast, the repulsive-only force field avoided such effects, making it more suitable for boiling simulations. The existence of the nonevaporative film was found to depend on the surface wettability. At lower wettability, the liquid film continuously evaporates, whereas at higher wettability, evaporation ceases once the film reaches a certain thickness.

The thickness of the nonevaporative film was found to increase with pressure. However, this growth diminishes at higher pressure levels, indicating a nonmonotonic relationship between pressure and film thickness. A novel boundary force field was developed and successfully applied to simulate boiling from thick films. This force field effectively maintained the liquid film near the boundary without causing noticeable changes in the liquid density. The interfacial thermal resistance was evaluated based on the liquid film depletion rate at 1 atm pressure, and the accommodation coefficient was calculated and found to be approximately 0.9 for various degrees of superheat. Further, the additional disjoining resistance has been calculated for finite film thickness and found to exhibit a minimum at 5–10 nm. The increase in this resistance beyond the range of film thickness may be related to the effect of near-interface density fluctuations on the evaporative process, which we have further elaborated upon with the help of VDOS analysis.

Future work will focus on exploring boiling dynamics in the presence of contaminants, such as secondary gases in the vapor phase or surfactants at the liquid–vapor interface, which are expected to significantly influence interfacial transport properties and boiling efficiency.

## Supplementary Material



## Notes

Selection of piston damping coefficient for faster equilibration; length-scale sensitivity of the repulsive-only force field (PDF)
