# Supplementary material for: Isobaric Molecular Dynamics Study of Liquid Film Boiling
Source: Langmuir. 2026 Feb 23;42(9):6959–70. doi: 10.1021/acs.langmuir.5c06528 (PMC12981021; doi:10.1021/acs.langmuir.5c06528)
Supplement: Supplementary file 1 [file la5c06528_si_001.pdf]

Supplementary information for

# Isobaric Molecular Dynamics Study of Liquid Film Boiling

Avik Saha and Omar K Matar*

Department of Chemical Engineering, Imperial College of London, London SW7 2AZ, UK

Number of pages: 2
Number of figures: 2

**Table of Contents**

**S1.** Selection of piston damping coefficient for faster equilibration ..................................................2
**S2.** Length-scale sensitivity of the repulsive-only force field: ..........................................................2

* O.matar@imperial.ac.uk

## S1. Selection of piston damping coefficient for faster equilibration

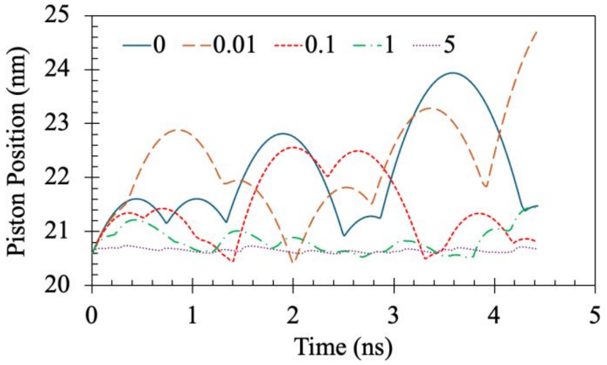


Figure S1: Piston oscillation with different damping coefficient

To optimise the damping coefficient, we simulated liquid block with a piston above it to maintain 1 atm pressure. Simulations are repeated with different values of $\zeta$ and the tracked the piston movement. In the Figure S1 we can see for $\zeta = 0$ and $\zeta = 0.01$, amplitude of the piston seems to rise with time. For, $\zeta = 0.1$ there are still oscillation, however, with time they seem to diminish. Beyond that value of $\zeta$ hardly any oscillations are observed, and the piston movement seems to be overdamped. So, we have selected $\zeta = 0.1$ as the optimum value of damping for the equilibration of the piston-based system.

## S2. Length-scale sensitivity of the repulsive-only force field:

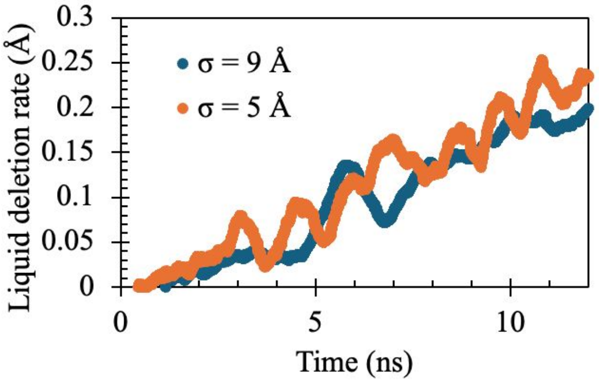


Figure S2: Comparison of the thick-film depletion rate for simulations using different length scales (σ = 9 Å and σ = 5 Å) for the repulsive-only piston–water force field.

Since we implement a novel repulsive-only force field between the piston wall and the water molecules, it is essential to examine the sensitivity of the results to the choice of the Lennard–Jones length-scale parameter σ for this interaction. To this end, we repeated the boiling simulation presented in Section 3.2 using σ = 5 Å, where we evaluated the liquid-film depletion rate for the thick-film case. The cutoff for this piston–water force field was also set to 5 Å, while the cutoffs for all other force fields were kept unchanged at 9 Å.

Figure S2 compares the liquid-film depletion rates obtained using σ = 9 Å (baseline case) and σ = 5 Å. Both simulations exhibit almost identical trends, with only minor fluctuations, demonstrating that the length-scale of this repulsive-only force field has negligible influence on the boiling characteristics.
